# 의사결정나무 분석을 이용한 한국 노인의 성별 근감소증 위험군 규명: 국민건강영양조사 자료 기반 횡단적 연구

**DOI:** 10.7586/jkbns.25.091

**Published:** 2026-02-27

**Authors:** Hee Sun Kim, Seok Hee Jeong, Se Young Jang

**Affiliations:** 1 College of Nursing · Research Institute of Nursing Science, Jeonbuk National University, Jeonju, Korea; 1 전북대학교 간호대학 · 간호과학연구소; 2 Division of Nursing, Jeonbuk National University Hospital, Jeonju, Korea; 2 전북대학교병원 간호부

**Keywords:** Aged, Sex, Sarcopenia, Decision trees, 노인, 성별, 근감소증, 의사결정나무

## Abstract

**Purpose:**

This study aimed to identify sex-specific sarcopenia risk groups among older adults.

**Methods:**

Data were obtained from 1,870 Korean adults aged 65 years or older who participated in the Korea National Health and Nutrition Examination Survey between 2022 and 2023. Sarcopenia was evaluated based on hand grip strength and skeletal muscle mass. Statistical analyses incorporating complex sample methods, along with decision tree analysis, were conducted using SPSS version 29.0.

**Results:**

The prevalence of reduced muscle strength, decreased muscle mass, and sarcopenia was 14.7%, 30.2%, and 8.1%, respectively. Sarcopenia was observed in 6.4% of men and 9.7% of women. In men, eleven sarcopenia risk pathways were identified based on age, anemia, education level, body mass index (BMI), perceived health status, hemoglobin A1c, diabetes, diastolic blood pressure, income, living status, acceptable macronutrient distribution range (AMDR) for protein, and aerobic exercise. The strongest predictor of sarcopenia risk in men was age ≥ 77 years, in combination with low educational attainment and anemia. In women, six sarcopenia risk pathways were identified based on age, BMI, AMDR for protein, high density lipoprotein-cholesterol, triglycerides, and living status. The strongest predictor of sarcopenia risk in women was age ≥ 74 years combined with low BMI.

**Conclusion:**

Sarcopenia risk factors and pathways differed substantially by sex among older adults. These findings suggest that sex-specific screening and targeted prevention strategies may be effective in reducing sarcopenia risk in older adults.

## 서론

### 1. 연구의 필요성

근감소증(sarcopenia)은 노화와 관련되어 있는 질환으로 연령과 관련된 근육 쇠약, 근육량 감소, 근력 저하, 신체 수행능력의 점진적이면서 전반적인 감소를 특징으로 한다[1]. 이러한 근감소증은 2016년 국제질병분류 제10차 개정판에 신규 질병코드로 등재되면서 질병으로 인식되고 고령화 사회에서 예방과 관리의 중요성이 강조되고 있다[2]. 노화가 진행됨에 따라 중년기 이후 약 10년 동안의 근육량은 평균 약 6.0%씩 감소한다고 보고되고 있으며[3], 전 세계적으로 노인의 약 10.0%~16.0%에서[4], 아시아의 노인 인구에서는 약 11.7%~25.0%가 근감소증을 진단받은 것으로 알려져 있다[5,6]. 노년층의 근감소증은 낙상 위험성 증가, 대사증후군 증가, 기능저하, 노인 우울 증가, 의료비 증가, 사망률 상승 등의 다양한 건강 관련 부정적인 결과와 밀접하게 관련이 있고[4], 고령화 사회에서 보건의료체계에 막대한 부담을 야기하며 최근 노인의 주요 건강문제로 인식되고 있다[4,7]. 그러므로, 근감소증 발생 위험이 높은 집단을 조기에 선별하고, 근감소증 발병에 기여하는 위험요인을 규명하는 것은 노인 건강증진을 위해 매우 중요하다[8].

2019년 유럽 근감소증 연구그룹(European Working Group on Sarcopenia in Older People, EWGSOP)의 개정된 진단기준에 의하면, 근감소증은 근력을 가장 중요한 근감소증의 지표로 삼고 있으며, 근육량이 같이 감소되어 있을 경우를 의미한다[1]. 2019년 아시아 근감소증 연구그룹(Asian Working Group for Sarcopenia, AWGS)에서는 근육량 감소와 더불어, 근력이나 근기능 중에 한 가지가 감소되어 있는 경우를 근감소증으로 정의하고 있다[9]. 근감소증 발생기전은 복합하고 다양한데, 노화로 인한 호르몬 변화, 신경근접합부의 기능 감소, 만성 염증 상태 등이 관련이 있는 것으로 보고되고 있다[10]. 이러한 생리적 변화는 성별에 따라 다르게 작용할 수 있는데, 염증, 단백질 항상성 변화, 미토콘드리아 기능 저하 등의 근감소증 기전이 남성과 여성에서 동일하게 작용하지만, 성별에 따라 변화의 크기나 반응의 민감성 차이가 있는 것으로 알려져 있다[11]. 노인 근감소증 관리로는 유산소 운동이나 저항운동, 신체활동 증진, 적정량 이상의 단백질 섭취, 절주, 만성질환 관리 등이 포함된다[5,8,10]. 그러나 노인 근감소증의 효과적인 관리를 위해 선행되어야 하는 것은 근감소증 발생에 영향을 미치는 다양한 위험요인을 파악하여 위험군을 선별하는 것이다. 노인의 근감소증 위험요인으로는 성별[12,13], 고령[12,14], 저체중[12,15], 낮은 교육수준[7], 혼자 사는 경우[7], 제한된 신체활동이나 좌식생활[1,15], 흡연[14], 우울[14], 골다골증[14], 영양장애[14,15], 과도한 수면시간[4], 당뇨병[16], 낮은 알부민과 헤모글로빈[16], 비만[1], 높은 저밀도 지단백 콜레스테롤(low density lipoprotein-cholesterol, LDL-C), 낮은 중성지방(triglyceride, TG)과 총콜레스테롤(total cholesterol, TC)[12] 등이 보고되고 있다.

한편, 근감소증 유병율은 성별에 따라 차이가 있는 것으로 알려져 있는데, 65~75세 연령층에서 여성노인이 남성노인보다 근감소증 유병율이 더 높은 것으로 보고되었다[14,17]. 근감소증 유발 요인은 성별 차이가 있는데[3], 남성노인의 경우 체질량지수(body mass index, BMI)와 허리둘레 등의 체성분과 대사 관련 요인이 주요 위험인자로 보고된 반면, 여성노인에서는 불량한 영양상태, 낮은 신체활동 수준, 비타민 D 결핍 등이 보다 밀접한 관련이 있었다[17]. 이에, 성별 특성을 고려한 근감소증 예방 및 관리 전략의 필요성이 제기되고 있다. 하지만, 선행연구들은 각각 위험요인별로 노인의 근감소증 간의 관계성을 개별적으로 살펴본 연구가 대부분이었고[3,7,17], 성별에 따라 여러 위험요인들이 상호 작용하며 근감소증 고위험군을 형성하는지에 대한 분석은 미흡한 실정이다. 기존 연구들 중 일부는 회귀분석을 통해 성별에 따른 근감소증 위험요인을 분석한 연구는 있었으나[7,13,17], 선행연구가 특정 연령층(65~74세)[17], 신체기능이 높은 65세 이상 집단[7], 또는 농촌 지역[13,18]에 국한되어 있어, 노인 인구 전체의 다양한 특성을 포괄적으로 설명하는데 한계가 있었다. 또한, 근감소증의 정의와 측정 지표로 골격근 지수만을 적용하거나[17], 근력 평가를 반영되지 않아 근감소증 정의의 다차원적 기준을 충분하게 고려하지 못한 한계가 있다. 더불어, 위험요인 변수가 주로 대상자의 인구학적·생활습관적 요인에 치중되어 있어[18,19], 영양상태, 신체활동 수준, 지질 대사 등의 생리적·대사적 요인을 충분히 반영하지 못하였다. 나아가, 대부분의 연구가 단일 변수 간의 독립적 관계 분석에 그쳐[20], 성별에 따라 여러 위험요인들이 어떻게 상호작용하며, 근감소증 위험군을 형성하는지에 대한 통합적 분석이 미비한 실정이다. 따라서, 이러한 기존 연구의 제한점을 보완하여 성별에 따라 근감소증 위험요인을 다차원적으로 분석할 수 있는 연구가 이루어질 필요가 있다. 이에, 대표성이 높고 다양한 인구사회학적·생리적 변수들이 포함된 국민건강영양조사 자료를 이용하여 다양한 변수들을 고려한 통합적 결과를 제시하는 분석방법을 활용한다면 근감소증 위험군을 보다 정교하게 분류할 수 있을 것으로 보인다.

따라서, 본 연구에서는 노인의 성별에 따른 근감소증 유병률의 차이를 파악하고, 근감소증 위험군을 규명하기 위한 모형을 구축하고자 한다. 이를 위해 성별에 따른 위험요인 간 상호작용을 시각적으로 탐색하고 위험군 분류기준을 도출함으로써 노인의 성별에 따른 근감소증 위험군을 명확하게 제시할 수 있는 의사결정나무(decision tree) 분석을 활용하고자 한다.

### 2. 연구의 목적

본 연구는 제9기(2022~2023년)의 국민건강영양조사 원시자료를 이용하여 한국 노인의 성별에 따른 근감소증 유병율을 파악하고, 근감소증 위험군을 규명하기 위함이다.

## 연구 방법

### 1. 연구 설계

본 연구는 국민건강영양조사를 활용한 이차자료 분석연구로, 65세 이상 노인의 근감소증 유병율과 위험요인을 파악하고, 성별에 따른 근감소증 위험군을 규명하기 위한 서술적 조사연구이다.

### 2. 연구 대상

본 연구대상자는 제9기 1차(2022년)와 2차(2023년) 국민건강영양조사에 참여한 65세 이상의 노인이다. 국민건강영양조사 제9기 1,2차 조사에 참여한 전체 표본 수는 총 13,294명이었다. 본 연구에서는 2019년 AWGS 가이드라인[9]에 따라서 근육량 및 근력이 감소한 상태를 근감소증으로 정의하였다. 전체 표본 중에서 65세 이상인 3,502명 중 근력 지표인 악력 미측정자(70명)와 근육량 지표인 골격근 지수를 측정하는 체성분검사 미측정자(614명)는 근감소증 진단이 불가능하여 분석에서 제외하였다. 또한, 최근 한 달간 와병 대상자 338명, 신체기능 중 활동제한이 있는 대상자 414명을 제외하였으며, 비정상적인 근감소 유발요인을 야기할 수 있는 질환인 만성신부전 진단받은 자 135명과 와병, 활동제한, 만성신부전 항목에 대한 결측값 61명을 제외하여 총 1,870명이 최종 분석대상자였다.

### 3. 연구 도구

본 연구에서는 기존 연구들[4,17,18]에서 노인의 근감소증 혹은 근력 감소 관련 요인으로 보고된 항목들을 기준으로 연구변수를 선정하였으며, 인구사회학적 및 질병관련 특성 11문항, 건강행태 특성 7문항, 생리적 지표 8문항, 영양상태 4문항, 악력, 골격근 지수, 근감소증 진단 여부로 구분하였다.

#### 1) 인구사회학적 및 질병관련 특성

인구사회학적 특성은 성별, 연령, 교육수준, 소득수준, 경제활동 상태, 동거상태를, 질병관련 특성은 만성질환 수, 고혈압, 당뇨병, 이상지질혈증 여부 및 빈혈 여부를 조사하였다. 구체적으로 교육수준은 ‘중학교 졸업 이하’, ‘고등학교 졸업’과 ‘대학교 졸업 이상’으로, 소득수준은 월평균 개인소득 ‘상’, ‘중’, ‘하’로 구분하였으며, 경제상태는 직업 여부로 ‘경제상태 군’과 ‘비경제상태 군’으로 재분류하였다. 동거상태는 세대유형 중 1인 가족을 ‘독거’로, 그 외의 유형을 ‘가족이나 혹은 친척과 같이 산다’로 구분하였다. 질병관련 특성은 총 만성질환 수는 고혈압, 당뇨병, 이상지질혈증을 진단받은 질환의 개수를 계산하여 ‘0개’, ‘1~2개’, ‘3개’로 분류하였다. 빈혈여부는 여성의 경우 헤모글로빈 수치가 < 12 g/dL, 남성은 < 13 g/dL인 경우로 ‘있음’으로 구분하였다.

#### 2) 건강행태

건강행태 특성은 흡연, 음주, 신체활동(유산소운동, 근력운동, 걷기), 좌식생활을 조사하였다. 흡연은 ‘현재 흡연’과 ‘과거 흡연 혹은 흡연한 적 없음’으로, 음주는 ‘전혀 안 마심 혹은 월 1회 미만’, ‘월 1잔 이상’으로 분류하였다. 유산소운동 실천 여부는 ‘중강도 신체활동을 주당 150분 이상 또는 고강도 신체활동을 주당 75분 이상 또는 중강도와 고강도 활동을 혼합하여 이에 상응하는 수준의 신체활동을 수행한 경우’로 정의하였다. 근력운동 실천자는 ‘최근 1주일 동안 팔굽혀 펴기 등의 근력운동을 주 2일 이상 실시한 경우’, 걷기운동 실천자는 ‘최근 1주일 동안 한 번에 10분 이상 걷기를 주 5일 이상 실천하고 하루 평균 30분 이상인 실시한 경우’로 구분하였다. 좌식생활은 하루 중 자는 시간을 제외하고 앉아 있거나 누워있는 총 시간으로, 좌식생활 시간이 7시간 이상인 경우 장시간 좌식생활군, 7시간 미만인 경우 단시간 좌식생활군으로 분류하였다. 주관적 건강상태는 ‘나쁨’, ‘보통’, ‘좋음’으로 구분하였다.

#### 3) 생리적 지표

생리적 지표는 BMI, 수축기혈압(systolic blood pressure, SBP), 이완기혈압(diastolic blood pressure, DBP), 당화혈색소(glycated hemoglobin), TG, TC, LDL-C, 고밀도 콜레스테롤(high density lipoprotein-cholesterol, HDL-C)을 이용하였다. BMI는 저체중(< 18.5 kg/m^2^), 정상(18.5~ < 25.0 kg/m^2^), 비만(≥ 25.0 kg/m^2^)으로 분류하였으며, 혈압은 2차와 3차 측정된 SBP와 DBP 평균값을 적용하였다. 고혈압 전단계 진단기준에 따라[21] SBP는 130 mmHg 미만과 이상으로, DBP는 80 mmHg 미만과 이상으로 재분류하였다. 혈액검사에서 정상 범위 수치를 활용하여, 당화혈색소는 6.5% 미만[22], TG는 150 mg/dL 미만, 그리고 TC는 200 mg/dL 미만, LDL-C는 130 mg/dL 미만, HDL-C는 40 mg/dL 이상을 정상으로 구분하였다[23].

#### 4) 영양상태

설문조사 2일 전 24시간 동안 섭취한 식품을 대상으로, 개인별 24시간 회상조사 방식을 이용하였고, 국가표준식품성분표 제10 개정판[24]을 사용하여 영양성분을 분석하였다. 이를 통해 계산된 에너지 섭취량(kcal)과 단백질 섭취량(g)을 각각 변수로 사용하였다. 단백질 섭취지표는 2020 한국인 영양소 섭취 기준을 근거로 산출하였다. 개인의 체중당 하루 단백질 섭취량(g/kg/day)으로 산출한 후, 평균 필요량(estimated average requirement) 기준에 따라 0.73g/kg/day 미만과 이상으로 구분하였다. 또한, 단백질 권장 섭취량(recommended nutrient intake, RNI)을 기준으로 0.91g/kg/day 미만과 이상으로 구분하였다[24,25]. 한편, 단백질 섭취량(g)에 4 kcal/g을 적용하여 총 에너지 섭취량 대비 단백질 비율을 산출하였고, 한국인 영양소 섭취기준에서 제시한 단백질 에너지 적정비율(acceptable macronutrient distrubution range, AMDR) 7.0%~20.0%를 만족한 경우와 이를 만족하지 못한 경우(< 7.0%, > 20.0%)로 구분하였다[24,25].

#### 5) 근력, 근육량 및 근감소증

근력검사는 디지털 악력계(T.K.K. 5401 Digital Grip Strength Dynamometer, Takei Scientific Instruments Co., Ltd., Niigata, Japan)를 이용하여 악력을 측정하였다. 2회 측정한 한손 또는 양손 악력값 중 주로 사용하는 손의 최댓값을 사용하였다. 이 값이 남성에서 28 kg 미만, 여성에서 18 kg 미만[9]인 경우를 근력저하로 정의하였다. 근육량은 임피던스체지방측정기(InBody 970 Body Composition Analyzer, InBody Co., Ltd., Seoul, Korea)로 측정하였다. 각 사지의 근육량을 더해 사지 근육량값을 구하고, 이를 키(m)의 제곱으로 나누어 골격근 지수(skeletal muscle index, SMI)를 산출하여 SMI가 남성에서 7.0 kg/m^2^ 미만, 여성에서 5.7 kg/m^2^ 미만[9]인 경우를 근육량 감소로 정의하였다. 이에, 근육량 저하가 있으면서 동시에 근력 감소가 있는 경우를 근감소증으로 정의하였다.

### 4. 자료 수집

본 연구는 제9기(2022~2023년) 국민건강영양조사 자료를 이용하였다. 이 자료에서 설문조사 표본은 다단계 층화집락 표본 추출법에 의해 선정되었다. 설문조사는 일대일 면접 혹은 자기기입식 방법으로 이루어졌으며, 대상자의 건강 설문조사, 혈액검사, 신체계측 등이 수행되었다. 자료는 국민건강영양조사 홈페이지(https://knhanes.kdca.go.kr/knhanes/main.do)에서 사용자 정보 등록 및 이용승인을 받은 후 다운로드하여 분석에 활용하였다.

### 5. 자료 분석

본 연구자료는 국민건강영양조사 원시자료분석 지침과 SPSS WIN 29.0 (IBM Corp., Armonk, NY, USA) 프로그램을 이용하여 분석하였다. 분석 시 복합표본 설계 정보(층화 strata, 집락 cluster, 가중치 weight)를 반영하였으며, 2개년도 평균 가중치를 적용하여 실수와 백분율, 평균과 표준편차를 산출하였다. 결측값은 유효한 값으로, 실수는 가중되지 않는 빈도로, 백분율과 평균과 표준오차는 가중치를 반영하여 산출하였다. 성별에 따른 근감소증과 대상자 특성 차이는 복합표본 교차분석, 복합표본 t-검정으로 비교하였으며, 통계적 유의수준은 *p* < .050을 기준으로 하였다. 본 연구에서는 65세 이상 노인의 근감소증 위험군을 규명하기 위해 의사결정나무 기반의 classification and regression tree (CRT) 알고리즘을 적용하였는데[26], 의사결정나무 분석 시에는 SPSS 통계 프로그램의 구조적 한계로 인해 복합표본 설계를 반영할 수 없어 비가중(unweighted) 방식으로 분석하였다. 의사결정나무의 CRT는 부모노드로부터 2개의 자식노드로 이진 분할을 반복하여 노드 내 동질성을 극대화하는 방법으로, 본 연구에서는 Gini 지수를 불순도 척도로 사용하였다. 의사결정나무 모형 구축 시 최대 분할수준은 5로 제한하였으며, 부모노드와 자식노드의 최소 사례수는 각각 10과 5로 설정하였다. 분할기준의 최소 기준은 0.0001로 설정하였으며, 모형의 안정성과 예측력을 검증하기 위해 10-fold 교차타당성 평가를 실시하였는데, 이는 구축된 모형예측력을 평가하는 과정을 총 10회 반복하여 평균 위험추정치 10개를 도출하고, 이를 전체 자료로 구축한 모형의 위험추정치와 비교하는 과정이다[26]. 또한, 의사결정나무 분석을 이용하여 구축한 예측모형의 성능 평가는 혼동행렬(confusion matrix)을 작성하였으며, 이를 바탕으로 정확도(accuracy), 민감도(sensitivity), 특이도(specificity)를 산출하였다.

### 6. 윤리적 고려

본 연구는 국민건강영양조사 원시자료를 이용한 2차 분석한 연구이다. 공개된 원시자료를 활용하기 전에, 국민건강영양조사 자료 이용자 준수사항과 보안서약을 이행하였으며, 연구에 사용된 자료는 개인식별 정보가 제거된 비식별화된 데이터로서 대상자의 익명성과 기밀성을 유지하였다. 본 연구수행 전 전북대학교 생명윤리심의위원회에서 심의면제 승인을 받았다(JBNU 2025-11-009).

## 연구 결과

### 1. 대상자의 근감소증 여부에 따른 일반적 특성 차이

본 연구대상자 중 근력 저하군은 14.7%, 근육량 저하군은 30.2%였으며, 근력과 근육량 모두 저하된 근감소증군은 8.1%로 나타났다. 대상자의 근감소증 여부에 따른 인구사회학적 및 질병관련 특성 차이는 성별(*p* = .017), 연령(*p* < .001), 교육수준(*p* < .001), 경제활동 상태(*p* = .030), 동거상태(*p* < .001), 고혈압(*p* = .037), 빈혈(*p* < .001)에 따라서 통계적으로 유의한 차이가 있었다(Table 1). 근감소증 집단별 유병율을 비교한 결과, 여성(9.7%), 75~80세 미만(11.3%)과 80세 이상(22.0%), 중학교 졸업 이하군(10.5%), 비경제활동 군(9.5%), 독거군(13.3%), 고혈압군(9.3%), 빈혈군(13.5%)에서 상대적으로 높게 나타났다.

대상자의 근감소증 여부에 따른 건강행태 차이로는 음주(*p* < .001), 유산소 운동(*p* = .013), 근력 운동(*p* < .001), 좌식생활(*p* < .001)에 따라서 통계적으로 유의한 차이가 있었다(Table 1). 구체적으로 근감소증 집단별 유병율을 비교한 결과, 평생 비음주자 혹은 월 1회 미만으로 음주하는 군(10.4%), 유산소 운동 미실천자(9.7%), 근력운동 미실천자(9.7%), 7시간 이상의 좌식생활군(9.3%)에서 상대적으로 높게 나타났다.

대상자의 근감소증 여부에 따른 생리적 차이로는 BMI (*p* < .001), 당화혈색소 (*p* = .011), TC (*p* = .033)에 따라서 통계적으로 유의한 차이가 있었다(Table 1). 근감소증 집단별 유병율을 비교한 결과, 저체중군(27.9%), 당화혈색소 6.5% 이상 군(12.0%), TC 200 mg/dL 미만 군(8.8%)에서 상대적으로 높게 나타났다.

대상자의 근감소증 여부에 따른 영양상태 차이로는 에너지 섭취량(*p* < .001), 단백질 섭취량(*p* < .001)에 따라서 통계적으로 유의한 차이가 있었다(Table 1). 근감소증 집단별 유병율을 비교한 결과, 에너지 섭취량이 낮은 군(10.0%)과 단백질 섭취량이 낮은 군(90.6%)에서 상대적으로 높게 나타났다.

### 2. 남성노인에서 근감소증 여부에 따른 일반적 특성 차이

본 연구대상자 중 남성노인은 47.4%이었으며, 이 중 근력 저하군은 47.8%, 근육량 저하군은 23.9%, 이 둘 모두 저하된 근감소증군은 6.4%였다. 남성노인에서 근감소증 여부에 따른 인구사회학적 및 질병관련 특성 중 연령(*p* < .001), 교육수준(*p* < .001), 동거상태(*p* = .033), 당뇨(*p* = .015), 빈혈(*p* < .001)에서 통계적으로 유의한 차이가 있었다(Table 2). 근감소증 집단별 유병율을 비교한 결과, 80세 이상(17.2%), 중학교 졸업 이하군(10.1%), 독거군(10.9%), 당뇨군(9.3%), 빈혈군(16.6%)에서 상대적으로 높게 나타났다.

건강행태 특성 중에서는 음주(*p* < .001), 유산소 운동(*p* < .001), 근력운동(*p* = .030), 좌식생활(*p* = .004)에서, 생리적 특성 중 BMI (*p* < .001), 당화혈색소(*p* < .001), TC (*p* = .018) 및 지각된 건강상태(*p* = .004)에서 통계적으로 유의한 차이가 있었다(Table 2). 근감소증 집단별 유병율을 비교한 결과, 평생 비음주자 혹은 월 1회 미만으로 움주하는 군(9.7%), 유산소 운동 미실천자(8.9%), 근력 운동 미실천자(7.9%), 7시간 이상의 좌식생활군(7.5%), 저체중(18.2%), 당화혈색소 6.5%이상 군(11.5%), TC 200 mg/dL 미만 군(7.4%), 주관적 건강상태가 좋지 않은 군(12.5%)에서 상대적으로 높게 나타났다.

대상자의 근감소증 여부에 따른 영양상태 차이로는 에너지 섭취량(*p* < .001), 단백질 섭취량(*p* = .013)에서 통계적으로 유의한 차이가 있었다(Table 1). 근감소증 집단별 유병율을 비교한 결과, 에너지 섭취량이 낮은 군(8.8%), 단백질 섭취량이 낮은 군(7.8%)에서 상대적으로 높게 나타났다(Table 2).

### 3. 여성노인에서 근감소증 여부에 따른 일반적 특성 차이

본 연구대상자 중 여성노인은 52.6%이었으며, 이 중 근력 저하군은 60.5%, 근육량 저하군은 29.0%, 이 둘 모두 저하된 근감소증군은 9.7%였다. 여성노인에서 근감소증 여부에 따른 인구사회학적 및 질병관련 특성 중 연령(*p* < .001), 동거상태(*p* = .011)에서 통계적으로 유의한 차이가 있었다(Table 2). 근감소증 집단별 유병율을 비교한 결과, 80세 이상(26.0%), 독거군(14.2%)에서 상대적으로 높게 나타났다.

건강행태 특성 중에서는 음주(*p* = .049), 근력운동(*p* < .001), 좌식생활(*p* = .042)에서, 생리적 특성 중 BMI (*p* < .001)에서 통계적으로 유의한 차이가 있었다(Table 2). 근감소증 집단별 유병율을 비교한 결과, 평생 비음주자 혹은 월 1회 미만으로 음주하는 군(10.7%), 근력운동 미실천자(10.9%), 7시간 이상의 좌식생활군(10.9%), 저체중군(40.3%)에서 상대적으로 높게 나타났다.

영양상태 특성 중 에너지 섭취량(*p* < .001), 단백질 섭취량(*p* < .001), 단백질 권장 섭취량 RNI (*p* = .039)에서 통계적으로 유의한 차이가 있었다. 근감소증 집단별 유병율을 비교한 결과, 에너지 섭취량이 낮은 군(10.6%), 단백질 섭취량이 낮은 군(10.4%), RNI 미만군(< 0.91g/kg/day) (11.2%)에서 상대적으로 높게 나타났다(Table 2).

### 4. 대상자의 성별에 따른 근감소증 위험군 분류

**Figure 1.**
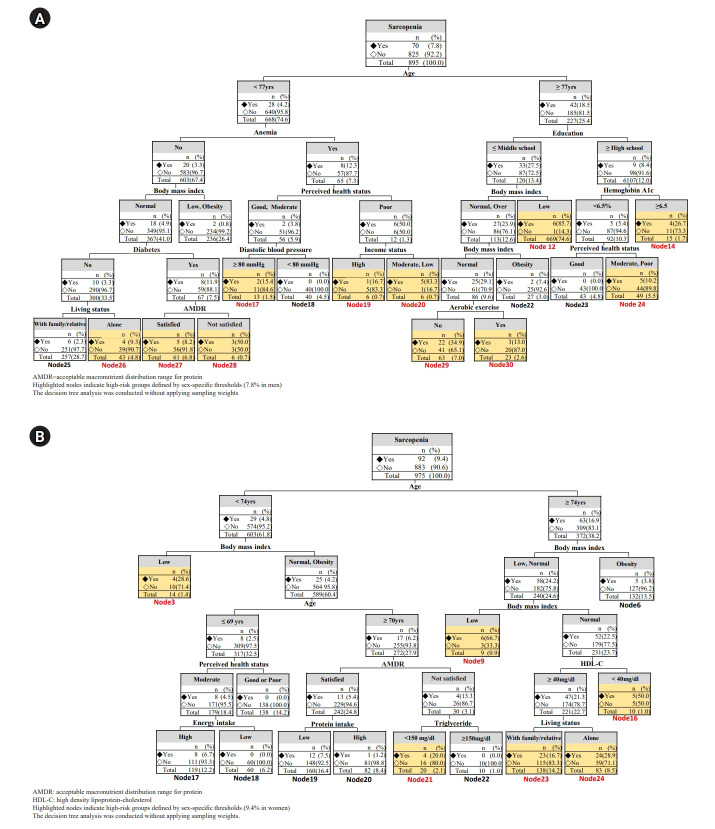
Decision trees illustrating sarcopenia risk groups among older men (A) and older women (B).

본 연구에서는 국내 65세 이상 노인의 성별에 따른 근감소증 위험군을 규명하기 위하여 남성노인과 여성노인을 대상으로 각각 의사결정나무 분석을 실시하였다(Figure 1-A, Figure 1-B). 본 연구에서 ‘근감소증 위험군’은 각 의사결정나무 분석에 투입된 전체 대상자의 분포를 나타내는 뿌리노드에서의 ‘근감소증 군의 비율’보다 해당 노드의 ‘근감소증 군의 비율이 높은 경우'로 정의하였다. 이에, 의사결정나무의 각 끝 노드에서 ‘근감소증의 비율’이 남성은 7.8%보다 높은 경우, 여성은 9.4%보다 높은 경우를 ‘근감소증 위험군’으로 규정하였다(Table 3).

분석 결과, 남성노인에서는 총 12개의 분류요인(연령, 빈혈, 교육, BMI, 주관적 건강인지, 당화혈색소, 당뇨병 진단, DBP, 소득, 동거상태, 단백질 에너지 적정 비율, 유산소 신체활동)이 분지를 형성하였고, 16개 끝노드 중 11개가 근감소증 위험군으로 분류되었다(Table 3, Figure 1-A). 첫 번째 위험군(노드 12)은 77세 이상·중졸 이하·저체중으로 근감소증 비율이 85.7%였다. 두 번째(노드 20)는 77세 미만·빈혈 있음·주관적 건강인지 불량·소득 수준 중·하위군으로 83.3%, 세 번째(노드 28)는 77세 미만·빈혈 없음·정상체중·당뇨병 있음·단백질 에너지 섭취 불만족군으로 50.0%였다. 네 번째(노드 29)는 77세 이상·중졸 이하·정상체중·유산소 신체활동 미실천군으로 34.9%, 다섯 번째(노드 14)는 77세 이상·고졸 이상·당화혈색소 6.5% 이상군으로 26.7%였다. 여섯 번째(노드 19)는 77세 미만·빈혈·건강인지 불량·소득 수준 상인 군으로 16.7%, 일곱 번째(노드 17)는 77세 미만·빈혈·건강인지 보통/좋음, DBP 80 mmHg 이상군으로 15.4%였다. 여덟 번째(노드 30)는 77세 이상·중졸 이하·정상체중·유산소 신체활동 실천군으로 13.0%, 아홉 번째(노드 24)는 77세 이상·고등학교 졸업·당화혈색소 6.5% 미만·건강인지 보통/불량군으로 10.2%였다. 열 번째(노드 26)는 77세 미만·빈혈 없음·정상체중·당뇨병 없음·독거군로 9.3%, 열한 번째(노드 27)는 77세 미만·빈혈 없음·정상체중·당뇨병 있음·단백질 에너지 섭취 만족군으로 근감소증 비율이 8.2%였다.

여성노인에서는 총 9개의 분류요인(연령, BMI, 주관적 건강인지, 단백질 에너지 적정비율, HDL-C, 에너지 섭취량, 단백질 섭취량, TG, 동거상태) 중 주관적 건강인지, 에너지 섭취량 및 단백질 섭취량을 제외한 6개 요인이 위험군 형성에 관여하였고, 13개 끝노드 중 6개가 근감소증 위험군으로 규명되었다(Table 3, Figure 1-B). 첫 번째 위험군(노드 9)은 74세 이상·저체중으로 근감소증 비율이 66.7%였고, 두 번째(노드 16)는 74세 이상·정상체중·HDL-C < 40 mg/dL군으로 50.0%였다. 세 번째(노드 24)는 74세 이상·정상체중·HDL-C ≥ 40 mg/dL·독거군으로 28.9%, 네 번째(노드 3)는 74세 미만·저체중군으로 28.6%였다. 다섯 번째(노드 21)는 74세 미만·저체중·단백질 에너지 섭취 불만족·TG < 150 mg/dL군으로 20.0%, 여섯 번째(노드 23)는 74세 이상·정상체중·HDL-C ≥ 40 mg/dL·가족/친척 동거군으로 16.7%의 근감소증 비율을 나타냈다.

본 연구에서 남성노인의 근감소증 위험군 규명을 위하여 구축된 모형의 위험추정값은 .07이었고, 10-fold 교차타당성 검증을 통한 평균 위험추정값은 .08로 거의 차이가 없었다. 여성노인의 경우에도 위험군 규명을 위하여 구축된 모형의 위험추정값은 .09, 10-fold 교차타당성 검증결과의 평균 위험추정값은 .10로 거의 차이가 없었다. 이에, 남성노인과 여성노인의 근감소증 위험군 규명을 위한 모형의 안정성이 확보되었다. 본 연구의 남성노인 근감소증 위험군 규명을 위한 모형의 정확도는 93.2%, 민감도는 15.7%였고, 특이도는 99.8%로 나타났으며(Appendix 1), 여성노인 근감소증 위험군 규명을 위한 모형의 정확도는 90.9%, 민감도는 12.0%였고, 특이도는 99.1%로 나타났다(Appendix 2).

## 논의

본 연구는 한국 노인의 성별에 따라 근감소증 유병 정도에 차이가 있는지를 확인하고, 성별에 따른 근감소증 위험군을 규명하기 위하여 시도되었다. 본 연구결과, 한국 노인의 근력 감소군은 14.7%, 근육량 감소군 30.2%, 근감소증군은 8.1%로 나타났다. 이러한 결과는 기존 선행연구에서 AWGS 기준을 적용한 국내 지역사회 노인 연구에서 보고된 6.8%~10.7% 수준의 근감소증 유병율과 유사한 양상을 보였다[24,27,28]. 반면 고령층이 비율이 높거나 보행속도 등의 근육 기능지표를 엄격히 포함한 연구에서는 20.0% 이상으로도 보고되어 본 연구보다 높은 경향을 나타났다[4]. 또한, 기능적 근감소증을 포함한 한국형 진단기준 (Korean Working Group on Sarcopenia)을 적용한 최근 연구에서는 남녀 각각 32.9%, 21.5%로 제시되어[28], 대상자 특성과 진단기준에 따라 차이가 있음을 시사하고 있다[29]. 한편, 선행연구 중 아시아 지역사회 노인의 경우 근감소증 분포가 13.6%~25.0%로 나타났는데, 이는 연구대상자가 고령 인구가 많고, 일부 연구에서 개정된 EWGSOP와 AWGS 2014 기준을 혼용하여 사용하였기 때문으로 해석된다[5,30]. 본 연구에서 성별로는 여성노인이 남성노인에 비해 근력(각각 60.5%, 47.8%)과 근육량(각각 29.0%, 23.9%) 모두 낮았고, 근감소증 유병율 또한 여성노인(9.7%)이 남성노인(6.4%)보다 높았다. 이는 기존 선행연구 결과들과 맥락을 같이 하는 것으로[6], 근감소증 진단과 관련된 성별 차이가 있는 것과 노인 인구 집단에서 성별이 근감소증 예방과 관리에 중요한 역할을 하고 있음을 시사하고 있다. 여성노인이 남성노인에 비해 근감소증이 더 발생하는 이유로는 폐경 이후의 근육 단백질 합성 저하와 산화 스트레스 증가 유발로 인하여 근력과 근육량의 감소를 가속화하는 것으로 알려져 있다[11]. 또한, 여성노인은 남성보다 신체활동 수준이 낮고 단백질 섭취량이 부족하여 근육량 저하가 두드러지는 경향이 있으며, 복부비만 및 인슐린 저항성과 같은 대사적 요인 증가하는 것으로 보고되어[17], 성별에 따라 근감소증 발생은 호르몬, 생활습관 등의 복합적인 요인이 작용한 결과로 보여진다.

본 연구결과에서 대상자의 일반적 특성 중 75세 이상의 연령, 중학교 졸업 이하의 저학력, 경제활동이 없는 상태, 혼자 사는 경우, 고혈압, 빈혈 등의 인구사회학적 및 질병관련 특성이나, 음주 경험이 없거나 월 1회 미만, 유산소 운동 미실천, 근력운동 미실천, 7시간 이상의 좌식생활 등의 건강행태 특성이나 저체중, 높은 당화혈색소, 낮은 콜레스테롤의 생리적 지표 그리고, 낮은 영양섭취량을 경험하는 군이 근감소증 유병과 밀접한 관련이 있는 것으로 나타나 이러한 위험한 특성들이 연계되어 노인의 근감소증에 영향을 미칠 수 있음을 시사하고 있다.

다음으로, 본 연구결과 한국 남성노인과 여성노인 각각의 근감소증 위험군을 규명하는 타당한 의사결정나무 모델이 제시되었다. 본 연구에서 근감소증 위험군은 성별 의사결정나무 분석에서 도출된 하위 집단 중, 전체 대상자에서 관찰된 근감소증 비율보다 높은 비율을 보이는 집단으로 정의하였고, 성별에 따라 상이한 근감소증 분포를 고려하여, 상대적으로 위험이 높은 집단을 규명하고자 하였다. 먼저 근감소증의 비율은 여성노인이 9.4%로 남성노인 7.8%에 비해 높은 비율이었으나 평균보다 근감소증 발생률이 높은 위험군의 수는 남성노인에서 11개 군, 여성노인에서 6개군으로 나타나 근감소증 평균 비율과 위험군의 수는 반대 양상을 보였다. 또한 남성노인의 근감소증 위험군 규명을 위한 모형에서는 총 12개의 연령과 같은 인구사회학적 특성, 당뇨병 등의 질병 상태, BMI, DBP와 같은 생리적 특성 및 영양상태, 신체활동 관련 요인들이 복잡하게 상호작용하여 근감소증 위험군을 규명하였다. 반면에 여성노인에서는 총 9개의 요인들이 근감소증 예측모형을 형성하고, 그 중 6개 요인인 연령, 동거상태, BMI, 단백질 에너지 적정비율, HDL-C, 중성지방의 상호작용을 통해 위험군이 규명되었는데, 이는 남성노인에 비하여 단순화된 양상을 보였으며, 연령과 BMI를 중심으로 지질 지표 및 동거 형태가 주요 위험요인으로 작용하였다. 이를 통해 동일한 한국 노인일지라도, 성별에 따라 근감소증 위험군이 매우 다른 양상을 보임을 확인할 수 있었으며, 이러한 차이는 본 연구의 매우 중요한 발견점이다. 또한 이는 일반적으로 단변량 분석에서 근감소증 발생에 미치는 영향을 각 변수마다 각각 분석한 결과로는 발견할 수 없는 의사결정나무 분석방법의 장점을 제시하고 있는 결과이기도 하다. 즉, 여성노인의 단변량 분석결과에서 여성노인의 근감소증 여부에 따라 HDL-C과 중성지방은 통계적으로 유의한 차이가 없는 것으로 나타나 이들은 여성노인 근감소증의 유의한 예측요인이 아닌 것으로 나타났다. 그러나 여러 요인들이 상호작용하는 의사결정나무 분석결과에서, HDL-C와 중성지방은 전체 여섯 개의 여성노인 위험군 중 네 개의 위험군을 선별하는 주요 요인으로 작용하고 있음을 확인할 수 있었다.

한편, 성별에 따라 근감소증 위험군 분류가 차이가 있었으나, 첫 번째 근감소증 위험군을 분류하는 공통 요인은 연령이었다. 연령이 증가함에 따라 근육 단백질 합성 감소, 미토콘드리아 기능 저하 및 산화스트레스 증가가 동반되며[31], 이는 근기능 저하와 밀접하게 연관되어 있다[1]. 또한, 연령이 증가함에 따라 신체활동 감소, 영양섭취 불균형, 사회적 고립 증가로 근감소증 발생위험이 높아질 수 있는데[14], 따라서 연령은 노인에서 근감소증 선별을 위한 1차 기준으로 활용가능한 핵심지표로 판단된다. 그러나, 연령이 동일하더라도 이후 분기 구조가 성별에 따라 다르게 전개된 점은, 근감소증 위험이 성별에 따라 서로 다른 경로와 조합으로 형성됨을 보여주고 있다. 즉, 남성노인 모형에서는 더 많은 변수와 위험군이 도출된 것은, 근감소증 고위험 집단이 단일한 유형이 아니라 질병 상태, 생리적 지표, 영양 및 신체활동 요인의 다양한 조합으로 구성된 여러 유형의 위험군으로 존재할 가능성을 보여준다. 반면 여성노인 모형은 연령과 BMI를 중심으로 위험군이 비교적 단순하게 분류되었으며, 지질대사 지표와 동거 상태는 특정 조건에서 위험군을 구분하는 보조적 요인으로 작용하였다. 이러한 구조적 차이는 근감소증 위험군의 형성 과정이 남성노인에서는 다차원적인 위험군 분류 변수들이 연결된 형태로, 여성노인에서는 노화와 체성분을 중심으로 한 비교적 집중된 경로로 나타날 수 있음을 보여주고 있으며, 이러한 결과는 성별에 따라 위험요인의 조합과 우선순위를 달리 고려할 필요성을 나타내고 있다.

다음으로, 각 성별에 따라 근감소증 위험군을 좀 더 자세히 논의해 보고자 한다. 먼저 남성노인은 총 11개의 위험군이 규명되었는데, 남성노인의 의사결정나무 모형에서 첫 번째 분지를 형성한 변인은 바로 연령이었다. 남성노인 연령 77세를 기준으로 총 11개의 위험군이 분지되었는데, 77세 미만에서 총 6개의 위험군, 77세 이상에서 5개의 위험군이 나타나 비슷한 양상을 보였다. 11개의 남성노인 위험군 중 근감소증 발생비율이 85.7%로 가장 높게 나타난 첫 번째 위험군은 노드 12로, 연령이 77세 이상인 중학교 졸업 이하 학력의 BMI가 저체중인 남성노인으로 나타났다. 즉, 고령으로 인한 생물학적 취약성과 더불어, 낮은 교육수준은 건강정보 이해도와 자기관리 능력, 적절한 영양섭취나 신체활동 실천에 어려움을 초래할 수 있으며, 이는 장기적으로 근육량과 근력 저하로 이어질 가능성이 있다[32]. 또한, 이러한 취약성은 체중 감소와 및 근육기능 저하와 동반될 수 있고, 특히 저체중(BMI)은 근감소증과 밀절하게 연관된 지표로 보고되고 있다[12,15]. 즉, 남성노인에서 근감소중 위험군을 선별할 때 연령, 교육수준, BMI와 같은 비교적 쉽게 파악 가능한 정보만으로도 고위험군을 식별할 수 있음을 보여준다. 그리고, 의사결정나무 모형을 통해 남성노인에서 총 11개의 위험군이 도출된 반면, 근감소증 발생률이 0.0%인 2개 군도 확인할 수 있었다. 바로 노드 18과 노드 23으로 노드 18은 일곱 번째 위험군인 노드 17(77세 미만·빈혈없음·건강인지 보통/좋음· DBP 80 mmHg 이상군)과 이완기 혈압수치만 80 mmHg미만으로 다를 뿐이며, 노드 23도 아홉 번째 위험군인 노드 24(77세 이상·고등학교 졸업·당화혈색소 6.5% 미만·주관적 건강인지 보통/불량군)와 주관적 건강인지가 좋다는 한 가지만 특성이 다를 뿐이다. 이러한 결과는 남성노인의 근감소증 위험군 분류 시에 시사하는 바가 있다. 즉, DBP 수치에 따라 동일한 특성을 가진 남성노인 집단에서도 근감소증 발생 비율에 차이가 있으며, 이는 혈압 상태가 남성노인의 근감소증 위험군 분류에서 중요한 구분 요인으로 작용할 수 있음을 시사한다. 본 연구결과는 고혈압과 노인에서의 근감소증 간 관련성이 있다는 선행연구와 맥락을 같이 하며[33], 혈압 수준이 골격근 미세혈관 기능 등과 연관되어 근감소증 위험과 연결될 가능성이 있음을 보여주고 있다[34]. 선행연구들에서 노인의 이완기혈압을 감소시킬 수 있는 효과적인 전략으로 자가관리 교육, 유산소 운동, 저항운동, 등척성 운동, 식이조절 등이 제시되고 있다[35]. 따라서 이러한 방법들을 임상현장 및 지역사회에서 남성노인들에게 적용해 봄으로써 이완기혈압 감소라는 일차적인 직접효과 뿐만 아니라 남성노인에서 근감소증 발생위험을 감소시키는 긍정적인 효과를 기대해 볼 수 있겠다. 또한 주관적 건강인지 수준에 따라서 근감소증 발생비율이 상이하게 나타난 점은, 남성노인의 근감소증 위험군 분류에서 주관적 건강인지가 의미있는 지표로 활용될 수 있음을 보여준다. 주관적 건강인지는 남성 노인에서 단일 심리적인 요인이라기보다는 기능저하, 통증, 활동제한, 만성질환 부담 등의 다양한 건강 취약성을 통합적으로 반영하는 지표로 작동했을 가능성이 높으며, 이는 실제로 낮은 지각된 건강상태가 근감소증 위험 예측요인으로 밝혀진 기존 연구와도 맥락을 같이한다[36]. 이처럼 남성노인의 근감소증을 규명할 수 있는 의사결정나무 모형은 여성노인에 비해 매우 다양한 요인들이 복잡하게 상호작용하고 있지만 본 연구를 통해 각 위험군의 특성이 규명된 만큼, 각 위험군을 선별함으로써 근감소증 발생 및 근감소증으로 인한 부정적인 결과들이 초래되지 않도록 의료진의 적극적인 선별과 개입이 요구된다.

다음으로 본 연구에서의 여성노인이 근감소증 위험군은 연령과 BMI를 중심으로, 영양섭취 지표 및 지질대사 지표, 독거 상태의 생활환경 요인이 결합되어 분류되어, 총 여섯 개의 위험군이 규명되었다. 그 중 여성노인의 의사결정나무 모형에서 첫 번째 분지를 형성한 변인은 남성과 동일하게 연령였다. 그러나 남성노인의 연령 컷 포인트가 77세인 반면 여성노인의 연령 컷 포인트는 74세로, 남성에 비해 여성노인이 3세가 적었다. 여성노인 연령 74세를 기준으로 74세 이상군에서는 총 여섯 개 위험군 중 다섯 개의 위험군이 나타났으며, 74세 미만에서는 단 한 개의 위험군이 나타났다. 이를 통해 여성노인 연령 74세가 여성노인이 근감소증 발생에서 갖는 중요한 분류요인임을 확인하였다. 그리고, 의사결정 나무 분석 결과 남성노인에 비해 여성노인에서 근감소증 위험이 더 이른 시점부터 증가할 수 있음을 보여주는데, 이는 폐경 이후의 호르몬 변화로 인한 근육 예비력 감소[37], 체성분 변화에 따른 저체중 노출 가능성 및 신체활동 패턴 변화 등이 누적되어 나타난 결과일 가능성을 보여주고 있다[37]. 즉, 여성노인의 근감소증 예방 및 선별은 남성노인보다는 상대적으로 이른 연령대부터 필요하다. 본 연구결과 여섯 개의 여성노인 위험군 중 근감소증 발생비율이 66.7%로 가장 높게 나타난 첫 번째 위험군은 노드 9였다. 노드 9는 연령이 74세 이상이며, BMI가 저체중인 그룹이다. 이는 남성노인의 첫 번째 위험군이 77세 이상인 중학교 졸업 이하 학력의 BMI가 저체중인 결과와 비교해보면, 남성노인에서는 연령, BMI 이외에도 학력이 중요한 분류요인으로 작용하였으나 이와 달리 여성노인에서 학력은 의사결정나무 모형이나 위험군을 선별하는데 관여되지 않아 남성노인과 차이를 보였다. 이는 의사결정나무 분석을 이용하여 한국 노인의 성별에 따른 건강관련 삶의 질 취약군을 규명한 선행연구[38]에서 한국 여성노인의 건강관련 삶의 질 취약군 분류에 교육수준이 중요 요인으로 제시된 것과는 차이가 있었다. 즉, 본 연구결과 연령과 BMI 두 가지 요인만으로도 여성노인에서 근감소증 발생률이 66.7%인 가장 높은 위험군을 선별해 낼 수 있다. 남성노인에 비해 전체적인 근감소증 유병율이 9.7%로 더 높은 여성노인을 대상으로 간단한 스크리닝만으로 가장 높은 근감소증 위험군을 선별해 낼 수 있다는 것은 간호현장에 유용한 지침을 제공한다.

다음으로 여성노인의 근감소증 위험군 여섯 개 중 유일하게 연령 74세 미만에서 분지한 유일한 위험군인 노드 3(네 번째 위험군)은 연령이 74세 미만이면서, BMI가 저체중인 그룹으로, 이 그룹 대상자의 28.6%가 근감소증으로 나타났다. 그런데 네 번째 위험군인 노드 3은 첫 번째 위험군인 노드 9와 동일하게 BMI가 저체중인 여성노인으로 구성되어 있으나, 연령 기준에서 차이를 보였다. 즉, BMI가 저체중인 여성노인들 중 연령이 74세 이상이면 근감소증 발생률이 66.7%인 가장 높은 위험군이 되는 반면, BMI가 저체중이어도 연령이 74세 미만이면 근감소증 발생률이 28.6%인 네 번째 위험군으로 분류되었다. 연령과 BMI가 상호작용하는 의사결정 나무분석 결과는 여성노인은 연령이 더 많은 그룹으로 갈수록 근감소증 발생비율이 더 높게 나타난 단변량 분석결과에서 제시할 수 없는 통합적인 결과를 제시해 준다는 의의가 있다. 또한 의사결정나무 모형을 통해 여성노인에서 총 여섯 개의 위험군이 도출된 반면, 근감소증 발생률이 0%인 세 개 군도 확인할 수 있었다. 바로 노드 12, 노드 18, 노드 22이다. 그 중에서도 노드 22는 다섯 번째 위험군인 노드 21과 중성지방 수치만 150 mg/dL 미만으로 다를 뿐이다. 본 연구에서, 중성지방 수치에 따라 동일한 특성을 가진 여성노인 집단에서도 근감소증 발생 비율에 차이가 관찰되었는데, 이는 노인환자에서 지질 지표(낮은 중성지방과 TC, 높은 LDL-C)가 근감소증과 유의하게 연관이 있다는 선행연구 결과와 맥락을 같이 한다[12], 그리고, 여성노인의 경우 지질대사 지표가 대사질환 위험보다는 에너지 섭취 및 영양상태를 반영하는 지표로 활용될 수 있음을 보여주고 있다. 본 연구결과에서 저체중 및 단백질 섭취부족과 결합된 상태에서 지질대사 지표의 변화가 나타난 점은, 여성노인의 근감소증 위험이 누적된 에너지 및 단백질 결핍 결과에 의해 야기될 수 있음을 보여주고 있다[17,39]. 따라서, 여성노인의 근감소증 예방 및 관리를 위해서는 의학적 접근뿐만이 아니라, 체중과 영양상태에 대한 정기적 사정, 단백질·에너지 섭취 증진을 위한 맞춤형 영양 교육, 독거 여성노인을 고려한 식사 지원 연계 등의 간호학적 중재가 병행될 필요가 있다.

종합해보면, 남성노인과 여성노인의 근감소증 위험군은 공통적으로 연령과 BMI가 핵심 요인이지만, 위험군 형성 양상은 성별 차이가 있었다. 남성노인의 위험군 형성은 77세를 기준으로 나누며, 대사질환, 영양, 사회경제적 취약성 및 신체활동 부족 등의 다차원적인 요인이 복합적으로 작용한 반면, 여성노인의 위험군 형성은 74세를 기준으로 나뉘며, 연령, BMI, 단백질 에너지 적정비율 및 지질대사 등이 밀접하게 연관되어 여성 노인의 근감소증이 상대적으로 노화과정과 대사적 요인에 의해서 결정될 수 있음을 제시하고 있다.

본 연구는 다음과 같은 제한점이 있다. 첫째, 본 연구에서의 의사결정나무 분석은 통계 프로그램의 구조적 특징으로 인해 복합표본 설계를 반영하지 못한 한계가 있다. 이에 따라 본 연구결과는 인과적 결론보다는 성별 근감소증 위험군 탐색을 위한 분석결과로 해석할 필요가 있다. 둘째, 의사결정나무 분석은 자료에 대한 적합도가 높아질수록 과적합(overfitting)의 가능성이 존재하며, 특히 표본 수가 상대적으로 적은 말단 노드에서는 분류 결과의 불안정성이 증가할 수 있다. 본 연구에서는 교차타당성 검증을 통해 모형의 안정성을 평가하였으나, 말단 노드 수준에서의 예측력은 표본 구성에 따라 달라질 수 있으므로 해석에 주의가 필요하다. 셋째, 본 연구에서 성별 근감소증 위험군 규명 모형의 민감도가 정확도와 특이성에 비해 상대적으로 낮게 분석된 바, 향후 연구에서는 위험군 판별 기준의 조정이나 Random Forest 등의 대안적 분석 모형 적용을 통해 민감도와 특이도의 균형을 개선할 필요가 있다. 넷째, 본 연구는 국민건강영양조사 자료를 활용하였기에 변수 및 도구 선정이 이미 확정되어 있어 추가적인 생물학적, 기능적 요인 등을 분석에 관심변수를 추가할 수 없었다는 제한점이 있다. 그럼에도 불구하고 신뢰성 있는 한국의 대단위 조사자료를 바탕으로 데이터마이닝 기법을 활용하여 한국 노인의 성별에 따른 근감소증 위험군을 규명하는 모델을 제시하고, 향후 성별 특성을 고려한 근감소증 선별 전략 수립을 위한 기초자료를 제시하였다는 점에서 의의가 있다.

## 결론

본 연구는 임상적 중요성이 대두되고 있는 근감소증에 대해 한국 노인을 대상으로 성별에 따른 근감소증 유병률의 차이를 제시하고, 성별에 따른 근감소증 위험군을 규명하는 의사결정나무 모형을 제시하였다. 이를 통해 먼저 선별적으로 파악되어야 하는 남성노인 근감소증 위험군 11개와 여성 근감소증 위험군 6개를 규명하였으며, 각 위험군을 구성하는 중요한 분류요인들의 상호조합을 파악하였다. 특히 본 연구결과를 통해 남성노인과 여성노인의 근감소증 위험군의 특성이 매우 다름을 발견하고, 지역사회 및 임상에서 근감소증 위험군의 선별 필요성과 대상자 맞춤형 예방전략의 필요성을 제시하였다.

본 연구결과를 통해 다음과 같이 제언하고자 한다. 첫째, 성별에 따른 한국 노인의 근감소증 위험을 선별할 수 있는 타당한 도구 및 간단한 어플리케이션 개발을 제언한다. 둘째, 성별에 따라 노인의 근감소증 발생을 예방할 수 있는 다양한 중재개발 및 그 효과를 검정하는 연구의 수행을 제언한다. 셋째, 의사결정나무뿐만 아니라 Random Forest, XGBoost 등 다양한 머신러닝/데이터마이닝 기법을 활용하여 위험군 분류를 위한 모델의 타당성과 일반화 가능성을 높이는 비교 연구가 필요함을 제언한다.

## Figures and Tables

**Table 1.** General Characteristics of Participants Stratified by Presence/Absence of Sarcopenia (N = 1,870)

Variables	Categories	Total (n = 1,870, 100.0%)	Sarcopenia (n = 162, 8.1%)	Normal (n = 1,708, 91.9%)	Rao-Scott χ² or Wald F (*p*)
		n^†^ (%^‡^)	n^†^ (%^‡^)	n^†^ (%^‡^)	
Sociodemographics and disease-related characteristics					
Sex	Men	895 (47.4)	70 (6.4)	825 (93.6)	6.93 (.017)
	Women	975 (52.6)	92 (9.7)	883 (90.3)	
Age (years)	65~69	729 (39.3)	25 (3.0)	704 (97.3)	96.27 (< .001)
	70~74	502 (24.6)	31 (6.0)	471 (94.0)	
	75~79	407 (23.4)	53 (11.3)	354 (88.7)	
	≥ 80	232 (12.7)	53 (22.0)	179 (78.0)	
	Mean ± SE^§^	72.05 ± 0.14	75.65 ± 0.40	71.73 ± 0.14	
Education^∥^	≤ Middle school	1,137 (58.9)	124 (10.5)	1,013 (89.5)	20.49 (< .001)
	High school	456 (25.0)	24 (4.5)	432 (95.5)	
	≥ College/University	275 (16.1)	14 (5.1)	261 (94.9)	
Income status^∥^	High	854 (49.3)	62 (6.7)	792 (93.3)	6.21 (.071)
	Moderate	375 (19.7)	32 (8.3)	343 (91.7)	
	Low	639 (31.0)	68 (10.3)	571 (89.7)	
Employment^∥^	Yes	818 (42.3)	54 (6.2)	764 (93.8)	7.25 (.030)
	No	1,049 (57.7)	108 (9.5)	941 (90.5)	
Living status	Alone	414 (20.9)	54 (13.3)	360 (86.7)	18.11 (< .001)
	With family/relative	1,456 (79.1)	108 (6.7)	1,348 (93.3)	
Comorbidities (number)	0	576 (30.4)	42 (6.3)	534 (93.7)	3.60 (.176)
	1~2	1,100 (59.4)	102 (8.8)	998 (91.2)	
	≥ 3	194 (10.2)	18 (9.3)	176 (90.7)	
Hypertension	Yes	978 (53.4)	93 (9.3)	885 (90.7)	4.41 (.037)
	No	892 (46.6)	69 (6.7)	823 (93.3)	
Diabetes	Yes	399 (21.3)	42 (9.7)	357 (90.3)	1.80 (.172)
	No	1,471 (78.7)	120 (7.7)	1,351 (92.3)	
Hyperlipidemia	Yes	808 (43.3)	63 (7.6)	745 (92.4)	0.50 (.536)
	No	1,062 (56.7)	99 (8.5)	963 (91.5)	
Anemia^∥^	Yes	255 (13.5)	39 (13.5)	216 (86.5)	11.24 (< .001)
	No	1,583 (86.5)	120 (7.2)	1,463 (92.8)	
Health behavior-related characteristics					
Smoking^∥^	None/ex-smoker	1,671 (89.9)	147 (8.3)	1,524 (91.7)	0.71 (.344)
	Current smoking	194 (10.1)	15 (6.6)	179 (93.4)	
Alcohol consumption^∥^	None or < 1 times/month	1,157 (62.1)	125 (10.4)	1,032 (89.6)	20.89 (< .001)
	≥ 1 times/week	708 (37.9)	37 (4.4)	671 (95.6)	
Aerobic exercise^∥^	Yes^¶^	669 (37.2)	41 (5.5)	628 (94.5)	10.30 (.013)
	No	1,189 (62.8)	121 (9.7)	1,068 (90.3)	
Muscle strength^∥^	Yes^#^	488 (23.8)	23 (3.9)	465 (96.1)	16.98 (< .001)
	No	1,380 (76.2)	139 (9.7)	1,241 (90.3)	
Walking^∥^	Yes^**^	888 (49.2)	72 (7.4)	816 (92.6)	1.30 (.388)
	No	979 (50.8)	89 (8.8)	890 (91.2)	
Sedentary time (hours)^∥^	Long (≥ 7)	1,183 (67.7)	116 (9.3)	1,067 (90.7)	8.762 (< .001)
	Short (< 7)	632 (32.3)	39 (5.3)	593 (94.7)	
	Mean ± SE	8.47 ± 0.12	8.37 ± 0.12	9.52 ± 0.32	
Perceived health status	Good	613 (34.1)	41 (6.2)	572 (93.8)	6.23 (.062)
	Moderate	944 (49.1)	83 (8.5)	861 (91.5)	
	Poor	313 (16.8)	38 (10.8)	275 (89.2)	
Physiological biomarker-related characteristics					
BMI (kg/m^2^)	Low (< 18.5)	54 (2.9)	17 (27.9)	37 (72.1)	60.11 (< .001)
	Normal (18.5–24.9)	1,168 (62.8)	130 (10.1)	1,038 (89.9)	
	Obesity (≥ 25.0)	648 (34.3)	15 (2.7)	633 (97.3)	
	Mean ± SE^§^	23.95 ± 0.08	21.83 ± 0.24	24.13 ± 0.08	
SBP (mmHg)^∥^	< 130	1,117 (59.1)	94 (7.7)	1.023 (92.3)	0.59 (.491)
	≥ 130	750 (40.9)	68 (8.7)	682 (91.3)	
	Mean ± SE^§^	127.57 ± 0.43	128.88 ± 1.75	127.46 ± 45	
DBP (mmHg)^∥^	< 80	1,418 (75.8)	131 (8.5)	1.287 (91.5)	1.41 (.271)
	≥ 80	449 (24.2)	31 (6.8)	418 (93.2)	
	Mean ± SE^§^	73.98 ± 0.21	71.67 ± 0.92	74.18 ± 0.22	
HbA1c (%)^∥^	< 6.5	1,540 (84.6)	120 (7.4)	1,420 (92.6)	6.99 (.011)
	≥ 6.5	294 (15.4)	39 (12.0)	255 (88.0)	
	Mean ± SE^§^	5.90 ± 0.02	6.00 ± 0.07	5.89 ± 0.02	
Triglycerides (mg/dL)^∥^	< 150	1,418 (76.5)	126 (8.3)	1,292 (91.7)	0.50 (.519)
	≥ 150	421 (23.5)	33 (7.3)	388 (92.7)	
	Mean ± SE^§^	123.47 ± 2.13	116.41 ± 6.14	124.09 ± 2.12	
Total cholesterol (mg/dL)^∥^	< 200	1,322 (72.3)	125 (8.8)	1.197 (91.2)	3.62 (.033)
	≥ 200	517 (27.7)	34 (6.1)	483 (93.9)	
	Mean ± SE^§^	177.06 ± 1.02	168.68 ± 2.84	177.80 ± 0.08	
LDL-C (mg/dL)^∥^	< 130	1,359 (74.1)	127 (8.7)	1,232 (91.3)	2.67 (.091)
	≥ 130	480 (25.9)	32 (6.3)	448 (93.7)	
	Mean ± SE^§^	104.87 ± 0.98	96.50 ± 2.60	105.61 ± 1.03	
HDL-C (mg/dL)^∥^	≥ 40	1,627 (88.8)	136 (8.0)	1,491 (92.0)	0.01 (.938)
	< 40	212 (11.2)	23 (8.2)	189 (91.8)	
	Mean ± SE^§^	55.95 ± 0.35	56.36 ± 1.32	55.91 ± 0.35	
Nutrition-related characteristics					
Energy intake (kcal)^∥^	High	623 (34.3)	28 (3.8)	595 (96.2)	26.04 (< .001)
	Low	1,194 (65.7)	127 (10.0)	1,067 (90.0)	
	Mean ± SE^§^	1683.29 ± 16.96	1396.80 ± 44.07	1707.72 ± 17.80	
Protein intake (g)^∥^	High	548 (30.4)	522 (95.7)	26 (4.3)	14.00 (< .001)
	Low	1,269 (69.6)	1,140 (90.6)	129 (9.4)	
	Mean ± SE^§^	62.43 ± 0.74	52.30 ± 1.96	63.29 ± 0.78	
Protein index (g/kg/day)^∥^	< EAR (< 0.73)	1,292 (72.0)	113 (8.0)	1,179 (92.0)	0.19 (.604)
	≥ EAR (≥ 0.73)	522 (28.0)	42 (7.4)	480 (92.6)	
	< RNI (< 0.91)	954 (53.2)	83 (8.3)	871 (91.7)	0.56 (.429)
	≥ RNI (≥ 0.91)	858 (46.8)	72 (7.4)	786 (92.6)	
AMDR for protein	Satisfied (7.0~20.0)	1,653 (87.6)	143 (7.9)	1,510 (92.1)	1.03 (.417)
	Not satisfied (< 7.0, > 20.0)	217 (12.4)	19 (9.8)	198 (90.2)	
Sarcopenia markers					
Hand grip strength	Decreased	283 (14.7)	162 (55.0)	121 (45.0)	955.15 (<. 001)
	Normal	1,587 (85.3)	-	1,587(100.0)	
	Mean ± SE^§^	28.16 ± 0.28	18.52 ± 0.42	29.01 ± 0.26	
Muscle mass	Decreased	570 (30.2)	162 (26.8)	408 (73.2)	381.47 (<. 001)
	Normal	1,300 (69.8)	-	1,300 (100.0)	
	Mean ± SE^§^	6.65 ± 0.03	5.61 ± 0.07	6.74 ± 0.03	

SE = Standard error; BMI = Body mass index; SBP = Systolic blood pressure; DBP = Diastolic blood pressure; HbA1c = Glycated hemoglobin; LDL-C = Low-density lipoprotein-cholesterol; HDL-C = High-density lipoprotein-cholesterol; EAR = Estimated average requirement for protein; RNI = Recommended nutrient intake for protein; AMDR = Acceptable macronutrient distribution range for protein.

† Non-weighted sample size;

‡ Weighted %;

§ Weighted mean & standard errors;

∥ Missing data;

¶ At least 150 minutes/week of moderate-intensity physical activity, at least 75 minutes/week of vigorous-intensity physical activity, or an equivalent combination of both;

# ≧ 2 days/week during the past week;

** Walked for at least 10 minutes at a time on ≧ 5 days/week, with an average of at least 30 minutes/day;

The analyses presented in this table were conducted using complex sample procedures with appropriate sampling weights applied.

**Table 2.** General Characteristics of Participants Stratified by Sex (N = 1,870)

Variables	Categories	Men (n = 895, 47.4%)		Rao-Scott χ² or Wald F (*p*)	Women (n = 975, 52.6%)		Rao-Scott χ² or Wald F (*p*)
		Sarcopenia (n = 70, 6.4%) n^†^ (%^‡^)	Normal (n = 825, 93.6%) n^†^ (%^‡^)		Sarcopenia (n = 92, 9.7%) n^†^ (%^‡^)	Normal (n = 883, 90.3%) n^†^ (%^‡^)	
Sociodemographic and disease-related characteristics							
Age (years)	65~69	10 (2.2)	328 (97.8)	38.68 (< .001)	15 (3.8)	376 (96.2)	56.79 (< .001)
	70~74	11 (4.5)	234 (95.5)		20 (7.5)	237 (92.5)	
	75~79	25 (10.4)	175 (89.6)		28 (12.0)	179 (88.0)	
	≥ 80	24 (17.2)	88 (82.8)		29 (26.0)	91 (74.0)	
Education^∥^	≤ Middle school	51 (10.1)	377 (89.9)	17.36 (< .001)	73 (10.7)	636 (89.3)	3.16 (.288)
	High school	11 (2.9)	258 (97.1)		13 (6.5)	174 (93.5)	
	≥ College/University	8 (3.8)	189 (96.2)		6 (8.5)	72 (91.5)	
Income status^∥^	High	23 (4.7)	383 (95.3)	4.59 (.061)	39 (8.5)	409 (91.5)	2.39 (.377)
	Moderate	15 (6.9)	163 (93.1)		17 (9.5)	180 (90.5)	
	Low	32 (8.6)	279 (91.4)		36 (11.8)	292 (88.2)	
Employment^∥^	Yes	26 (5.1)	412 (94.9)	2.12 (.248)	28 (7.3)	352 (92.7)	3.85 (.183)
	No	44 (7.5)	411 (92.5)		64 (11.1)	530 (88.9)	
Living status	Alone	13 (10.9)	110 (89.1)	4.36 (.033)	41 (14.2)	250 (85.8)	9.38 (.011)
	With family/relative	57 (5.7)	715 (94.3)		51 (7.8)	633 (92.2)	
Comorbidities (number)	0	20 (5.5)	284 (94.5)	0.74 (.636)	22 (7.2)	250 (92.8)	2.48 (.358)
	1~2	43 (6.7)	451 (93.3)		59 (10.6)	547 (89.4)	
	≥ 3	7 (7.7)	90 (92.3)		11 (10.8)	86 (89.2)	
Hypertension	Yes	29 (5.5)	382 (94.5)	0.81 (.245)	40 (7.7)	441 (92.3)	4.19 (.063)
	No	41 (7.0)	443 (93.0)		52 (11.5)	442 (88.5)	
Diabetes	Yes	23 (9.3)	188 (90.7)	3.85 (.015)	73 (9.5)	714 (90.5)	0.08 (.796)
	No	47 (5.5)	637 (94.5)		19 (10.2)	169 (89.8)	
Hyperlipidemia	Yes	51 (6.7)	548 (93.3)	0.42 (.516)	70 (6.4)	277 (93.6)	1.21 (.335)
	No	19 (5.6)	277 (94.4)		48 (10.8)	415 (89.2)	
Anemia^∥^	Yes	24 (16.6)	94 (83.4)	22.45 (< .001)	74 (9.3)	742 (90.7)	0.30 (.614)
	No	46 (4.9)	721 (95.1)		15 (10.8)	122 (89.2)	
Health behavior-related characteristics							
Smoking^∥^	None / ex-smoker	58 (6.6)	663 (93.4)	0.56 (.336)	89 (9.6)	861 (90.4)	2.44 (.155)
	Current smoking	12 (5.1)	162 (94.9)		3 (20.9)	17 (79.1)	
Alcohol consumption^∥^	None or < 1 times/month	42 (9.7)	330 (90.3)	11.07 (< .001)	83 (10.7)	702 (89.3)	4.68 (.049)
	≥ 1 times/week	28 (4.1)	495 (95.9)		9 (5.4)	176 (94.6)	
Aerobic exercise^∥^	Yes^¶^	15 (3.0)	343 (97.0)	12.52 (< .001)	26 (8.5)	285 (91.5)	0.82 (.477)
	No	55 (8.9)	476 (91.1)		66 (10.3)	592 (89.7)	
Muscle strength exercise^∥^	Yes^#^	16 (4.1)	314 (95.9)	4.76 (.030)	7 (3.5)	151 (96.5)	10.38 (.001)
	No	54 (7.9)	509 (92.1)		85 (10.9)	732 (89.1)	
Walking^∥^	Yes^**^	33 (6.5)	399 (93.5)	0.05 (.853)	54 (11.0)	464 (89.0)	2.20 (.177)
	No	36 (6.1)	426 (93.9)		39 (8.2)	417 (91.8)	
Sedentary time (hours)^∥^	Long (≥ 7)	50 (7.5)	505 (92.5)	4.05 (.004)	66 (10.9)	562 (89.1)	4.52 (.042)
	Short (< 7)	18 (3.9)	302 (96.1)		21 (6.5)	291 (93.5)	
Physiological biomarkers							
BMI (kg/m^2^)	Low (< 18.5)	7 (18.2)	24 (81.8)	18.64 (< .001)	10 (40.3)	13 (59.7)	46.94 (< .001)
	Normal (18.5-24.9)	58 (7.8)	519 (92.2)		72 (12.3)	519 (87.7)	
	Over (≥ 25)	5 (1.9)	282 (98.1)		10 (3.3)	351 (96.7)	
SBP (mmHg)^∥^	< 130	40 (6.0)	496 (94.0)	0.25 (.574)	54 (9.2)	527 (90.8)	0.39 (.596)
	≥ 130	30 (6.8)	329 (93.2)		38 (10.4)	353 (89.6)	
DBP (mmHg)^∥^	< 80	55 (6.8)	594 (93.2)	0.80 (.311)	76 (10.0)	693 (90.0)	0.29 (.669)
	≥ 80	15 (5.2)	231 (94.8)		16 (8.7)	187 (91.3)	
HgA1C (%)^∥^	< 6.5	49 (5.5)	686 (94.5)	7.02 (.001)	71 (9.0)	734 (91.0)	1.75 (.228)
	≥ 6.5	21(11.5)	127 (88.5)		18(12.6)	128 (87.4)	
Triglyceride (mg/dL)^∥^	< 150	57 (7.2)	605 (92.8)	2.53 (.197)	69 (9.2)	687 (90.8)	0.39 (.615)
	≥ 150	13 (4.2)	210 (95.8)		20 (10.7)	178 (89.3)	
Total cholesterol (mg/dL)^∥^	< 200	62 (7.4)	615 (92.6)	4.09 (.018)	63 (10.2)	582 (89.8)	1.11 (.280)
	≥ 200	8 (3.5)	200 (96.5)		26 (8.0)	283 (92.0)	
LDL-C (mg/dL)^∥^	< 130	61 (7.2)	622 (92.8)	2.62 (.092)	66 (10.1)	610 (89.9)	0.86 (.352)
	≥ 130	9 (4.1)	193 (95.9)		23 (8.1)	255 (91.9)	
HDL-C (mg/dL)^∥^	≥ 40	53 (6.2)	681 (93.8)	0.27 (.557)	83 (9.5)	810 (90.5)	0.04 (.829)
	< 40	17 (7.4)	134 (92.6)		6 (10.3)	55 (89.7)	
Perceived health status	Good	17 (3.9)	333 (96.1)	11.07 (.004)	24 (9.3)	239 (90.7)	0.07 (.964)
	Moderate	36 (6.9)	387 (93.1)		47 (9.9)	474 (90.1)	
	Poor	17 (12.5)	105 (87.5)		21 (9.8)	170 (90.2)	
Nutrition-related characteristics							
Energy intake (kcal)	High	23 (4.1)	414 (95.9)	9.55 (.002)	5 (3.0)	181 (97.0)	10.15 (.002)
	Low	45 (8.8)	384 (91.2)		82 (10.6)	683 (89.4)	
Protein intake (g)^∥^	High	20 (4.5)	342 (95.5)	4.62 (.033)	6 (3.9)	180 (96.1)	8.54 (.004)
	Low	48 (7.8)	456 (92.2)		81 (10.4)	684 (89.6)	
Protein index^∥^ (g/kg/day)	< EAR	52 (6.2)	618 (93.8)	0.21 (.578)	61 (10.0)	561 (90.0)	1.47 (.200)
	≥ EAR	16 (7.2)	179 (92.8)		26 (7.6)	301 (92.4)	
	< RNI (0.91)	35 (5.7)	468 (94.3)	1.16 (.257)	48 (11.2)	403 (88.8)	4.34 (.039)
	≥ RNI (≥ 0.91)	33 (7.5)	329 (92.5)		39 (7.3)	457 (92.7)	
AMDR for protein	Satisfied (7.0~20.0)	62 (6.3)	736 (93.7)	0.08 (.780)	81 (9.3)	774 (90.7)	0.94 (.463)
	Not satisfied (< 7.0, > 20.0)	8 (7.0)	89 (93.0)		11 (12.0)	109 (88.0)	
Sarcopenia markers							
Hand grip strength	Decreased	70 (47.8)	60 (52.2)	395.82 (< .001)	92 (60.5)	61 (39.5)	548.49 (< .001)
	Normal		765 (100.0)		-	765 (100.0)	
	Mean ± SE^§^	23.71 ± 0.47	35.78 ± 0.27	79.94 (< .001)	15.45 ± 0.22	22.69 ± 0.16	124.50 (< .001)
Muscle mass	Decreased	70 (23.9)	188 (76.1)	167.67 (< .001)	92 (29.0)	220 (71.0)	208.20 (< .001)
	Normal	-	637 (100.0)		-	663 (100.0)	

SE = Standard error; BMI = Body mass index; SBP = Systolic blood pressure; DBP = Diastolic blood pressure; HbA1c = Glycated hemoglobin; LDL-C = Low density lipoprotein-cholesterol; HDL-C = High density lipoprotein-cholesterol; EAR = Estimated average requirement; RNI = Recommended nutrient intake; AMDR = Acceptable macronutrient distribution range.

† Non-weighted sample size;

‡ Weighted %;

§ Weighted mean & standard errors;

∥ Missing data;

¶ At least 150 minutes/week of moderate-intensity physical activity or at least 75 minutes/week of vigorous-intensity physical activity or an equivalent combination of both;

# ≧ 2 days/week during the past week,

** Walked for at least 10 minutes at a time on ≧ 5 days/week, with an average of at least 30 minutes/day);

The analyses presented in this table were conducted using complex sample procedures with appropriate sampling weights applied.

**Table 3.** Decision-tree Analysis for Sarcopenia Groups (N = 1,870)

Sex	Group	Node ID	Total n (%)	Sarcopenia n (%)
Men	Risk Group ^†^	12	7 (0.8)	6 (85.7)
		20	6 (0.7)	5 (83.3)
		28	6 (0.7)	3 (50.0)
		29	63 (7.0)	22 (34.9)
		14	15 (1.7)	4 (26.7)
		19	6 (0.7)	1 (16.7)
		17	13 (1.5)	2 (15.4)
		30	23 (2.6)	3 (13.0)
		24	49 (5.5)	5 (10.2)
		26	43 (4.3)	4 (9.3)
		27	61 (6.8)	5 (8.2)
	Others		652 (69.3)	15 (0.0~7.4)
Women	Risk Group ^†^	9	9 (0.9)	6 (66.7)
		16	10 (1.0)	5 (50.0)
		24	83 (8.5)	24 (28.9)
		3	14 (1.4)	4 (28.6)
		21	20 (2.1)	4 (20.0)
		23	138 (14.2)	23 (16.7)
	Others		701 (71.9)	26 (0.0~7.5)

The decision tree analysis presented in this table was conducted without applying sampling weights.

† The high-risk group was defined as cases in which the proportion of sarcopenia exceeded 7.8% in men and 9.4% in women, respectively.

## Data Availability

The data supporting the findings of this study are available from the corresponding author upon reasonable request.

## Appendix 1. Accuracy of the Predictive Model for Sarcopenia in Men

**Table:**

		Prediction			Forecasting measures	%
		Yes (n)	No (n)	Total (n)		
Account	Yes	11	59	70	Accuracy	93.2
	No	2	823	825	Sensitivity	15.7
	Total	13	882	895	Specificity	99.8

## Appendix 2. Accuracy of the Predictive Model for Sarcopenia in Women

**Table:**

		Prediction			Forecasting measures	%
		Yes (n)	No (n)	Total (n)		
Account	Yes	11	81	92	Accuracy	90.9
	No	8	875	885	Sensitivity	12.0
	Total	19	956	975	Specificity	99.1
